# Impact of energy loss index on left ventricular mass regression after aortic valve replacement

**DOI:** 10.1007/s12574-013-0196-7

**Published:** 2013-11-26

**Authors:** Terumasa Koyama, Hiroyuki Okura, Teruyoshi Kume, Kenzo Fukuhara, Koichiro Imai, Akihiro Hayashida, Yoji Neishi, Takahiro Kawamoto, Kazuo Tanemoto, Kiyoshi Yoshida

**Affiliations:** 1 Division of Cardiology, Kawasaki Medical School, 577 Matsushima, Kurashiki, 701-0192 Japan; 2 Division of Cardiovascular Surgery, Kawasaki Medical School, Kurashiki, Japan

**Keywords:** Prosthesis–patient mismatch, Aortic valve replacement, Aortic stenosis, Energy loss coefficient, Energy loss index

## Abstract

**Background:**

Recently, the energy loss index (ELI) has been proposed as a new functional index to assess the severity of aortic stenosis (AS). The aim of this study was to investigate the impact of the ELI on left ventricular mass (LVM) regression in patients after aortic valve replacement (AVR) with mechanical valves.

**Methods:**

A total of 30 patients with severe AS who underwent AVR with mechanical valves was studied. Echocardiography was performed to measure the LVM before AVR (pre-LVM) (*n* = 30) and repeated 12 months later (post-LVM) (*n* = 19). The ELI was calculated as [effective orifice area (EOA) × aortic cross sectional area]/(aortic cross sectional area − EOA) divided by the body surface area. The LVM regression rate (%) was calculated as 100 × (post-LVM − pre-LVM)/(pre-LVM). A cardiac event was defined as a composite of cardiac death and heart failure requiring hospitalization.

**Results:**

LVM regressed significantly (245.1 ± 84.3 to 173.4 ± 62.6 g, *P* < 0.01) at 12 months after AVR. The LVM regression rate negatively correlated with the ELI (*R* = −0.67, *P* < 0.01). By receiver operating characteristic (ROC) curve analysis, ELI <1.12 cm^2^/m^2^ predicted smaller (<−30.0 %) LVM regression rates (area under the curve = 0.825; *P* = 0.030). Patients with ELI <1.12 cm^2^/m^2^ had significantly lower cardiac event-free survival.

**Conclusion:**

The ELI as well as the EOA index (EOAI) could predict LVM regression after AVR with mechanical valves. Whether the ELI is a stronger predictor of clinical events than EOAI is still unclear, and further large-scale study is necessary to elucidate the clinical impact of the ELI in patients with AVR.

## Introduction

Prosthesis–patient mismatch (PPM) was first described as a condition where the effective orifice area (EOA) of a normally functioning heart valve prosthesis is too small in relation to the patient’s body size, which results in high transvalvular pressure gradients [1]. Patients with PPM have worse functional class and exercise capacity and reduced regression of left ventricular (LV) hypertrophy after aortic valve replacement (AVR) compared with patients without PPM [2, 3]. Furthermore, PPM has been associated with increased incidence of late cardiac events [4–8].

Although the EOA derived from the continuity equation or direct planimetry of the stenotic aortic valve orifice were used to assess the severity of the aortic stenosis (AS) [9, 10], overestimation of the EOA could occur in the clinical setting because of the pressure recovery phenomenon [11, 12]. The Doppler-derived energy loss coefficient (ELCo) or energy loss index (ELI) has been proposed as a functional index to assess the severity of AS [11–13]. Although the ELCo or ELI may be related to left ventricular mass (LVM) regression after AVR with bioprosthetic valves [14], the impact of the ELI on LVM regression and clinical event after AVR with mechanical valves in patients with AS is unknown. Therefore, the objective of this study was to investigate the impact of the ELI on LVM regression in patients who underwent AVR with mechanical valves.

## Methods

### Patients

This study population included consecutive 30 patients (aged 62.8 ± 7.7 years; 15 men) with severe AS who underwent AVR with mechanical valves at our center between March 2002 and December 2010.

Indications for AVR were symptomatic severe AS (*n* = 20), asymptomatic severe AS with a high likelihood of rapid progression (*n* = 4), asymptomatic severe AS undergoing coronary artery bypass graft (CABG, *n* = 3), and extremely severe AS (peak aortic jet velocity >5.0 m/s, *n* = 3).

The prosthetic valves used in this study were the ATS (ATS Medical, Inc., Minneapolis, MN, USA) in 13 patients (valve size 19 mm, *n* = 4; valve size 21 mm, *n* = 2; valve size 23 mm, *n* = 6; valve size 25 mm, *n* = 1), the ATS AP (ATS Medical, Inc., Minneapolis, MN, USA) in 3 patients (valve size 18 mm, *n* = 2; valve size 24 mm, *n* = 1), the St. Jude Medical Standard (Medtronic, Minneapolis, MN, USA) in 3 patients (valve size 19 mm, *n* = 2; valve size 21 mm, *n* = 1), the St. Jude Medical Regent in 3 patients (valve size 19 mm, *n* = 2; valve size 21 mm, *n* = 1), the MCRI On-X valve (Medical Carbon Research Institute, LLC, Austin, TX, USA) in 3 patients (valve size 19 mm, *n* = 2; valve size 23 mm, *n* = 1), the Edwards Mira (Edwards Lifesciences, Irvine, CA, USA) in 1 patient (valve size 19 mm), and the Carbomedics Standard (Sulzer Carbomedics, Austin, TX, USA) in 4 patients (valve size 19 mm, *n* = 2; valve size 21 mm, *n* = 2). The study protocol was approved by the ethics committee of Kawasaki Medical School, and informed consent was given by each patient.

The presence of hypertension, hyperlipidemia, or diabetes mellitus was determined using the following criteria. Hypertension was defined as blood pressure >140/90 mmHg or current use of antihypertensive medication. Hyperlipidemia was defined as total cholesterol level >220 mg/dL or triglyceride level >150 mg/dL or current use of lipid lowering medication. Diabetes mellitus was defined as fasting plasma glucose level >126 mg/dL, plasma glucose level (at any time) >200 mg/dL, or current use of anti-diabetic medication. We excluded patients with systolic LV dysfunction before or after AVR (LV ejection fraction <30 %).

### Echocardiography

Echocardiographic examinations were performed before, 1 month, and 12 months after AVR. Echocardiographic parameters included the LV dimension, LV wall thickness, LV ejection fraction, and LVM. The LV dimension and LV wall thickness were measured using the two-dimensional method, and the LV ejection fraction was measured using the modified Simpson’s method [15]. The LVM was calculated using the method of Devereux et al. [16]. Changes in the LVM were assessed using both absolute LVM regression and the LVM regression rate. Absolute LVM regression (g) was calculated as post-LVM − pre-LVM. The LVM regression rate (%) was calculated as 100 × (post-LVM − pre-LVM)/pre-LVM [4]. The transvalvular gradients were measured using a continuous-wave Doppler technique. The pre-operative EOA was calculated according to the continuity equation. The EOA index (EOAI) was calculated as the EOA divided by the body surface area (BSA). The aortic diameter was measured at the level of the sinotubular junction [17]. The aortic cross sectional area (AA) was calculated as 3.14 × (aortic diameter/2)^2^. The ELCo was calculated as [EOA − AA]/(AA − EOA) [12, 13, 18]. The ELI was calculated as the ELCo divided by the BSA. Known EOA values for each prosthetic valve were used to calculate the ELCo [4, 12, 19–21]. The change in the EOAI (ΔEOAI) (cm^2^/m^2^) after AVR was calculated as post-operative EOAI − pre-operative EOAI. The change in the ELI (ΔELI) (cm^2^/m^2^) was calculated as post-operative ELI − pre-operative ELI [22].

A cardiac event was defined as a composite of cardiac death and heart failure requiring hospitalization.

### Statistical methods

All data were statistically analyzed using the SPSS statistical software (version 20.0, SPSS Inc., Chicago, IL, USA). Continuous variables were expressed as mean ± standard deviation and compared using a two-tailed paired Student’s *t*-test. Comparison between the two main groups was made with Fisher’s exact tests for categorical variables. For continuous variables, analysis of variance (ANOVA) with post hoc analysis using the Scheffé test was used to differentiate among the 3 groups of data. The relationship between the LVM regression rate and the EOAI or the ELI was evaluated by means of simple linear regression analysis to calculate *r* (Pearson’s correlation coefficient). Using receiver operating characteristic (ROC) curves (i.e., plots of sensitivity vs. 1− specificity), we defined the best cutoff value of the ELI for detecting patients with higher LVM regression rates after AVR and survival and freedom from cardiac events. A *P* value of less than 0.05 was considered significant.

## Results

The baseline clinical characteristics are summarized in Table 1. Twenty-six of 30 patents had symptoms related to severe AS. Echocardiographic findings before, 1 month, and 12 months after AVR are shown on Table 2. Eleven of 30 patients had no echocardiographic data at 12 months because they were followed at other hospitals without routine echocardiographic examinations. The LV diastolic diameter, interventricular septal thickness, posterior wall thickness, and LVM significantly decreased. The mean values of absolute LVM regression and the LVM regression rate from before AVR to 12 months after AVR were −76.8 ± 37.9 g and −30.0 ± 9.26 %, respectively. After AVR, the peak aortic velocity and mean pressure gradient decreased significantly (Table 3).

**Table 1:** Clinical characteristics

	Total (*n* = 30)
Age (years)	62.8 ± 7.7
Male gender [*n* (%)]	15 (50)
Body surface area (m^2^)	1.58 ± 0.21
Atrial fibrillation (%)	5 (17)
Symptoms	
Angina [*n* (%)]	5 (17)
Syncope [*n* (%)]	1 (3)
Heart failure [*n* (%)]	20 (67)
Hypertension [*n* (%)]	21 (70)
Dyslipidemia [*n* (%)]	13 (43)
Diabetes mellitus [*n* (%)]	10 (33)
Smoking [*n* (%)]	10 (33)
Hemodialysis [*n* (%)]	11 (37)
Coronary artery disease [*n* (%)]	9 (30)

**Table 2:** Pre- and post-operative (1 and 12 months) echocardiographic findings

	Pre-AVR (*n* = 30)	Post-AVR (1 month) (*n* = 26)	Post-AVR (12 months) (*n* = 19)	*P* value
LVDd (cm)	5.03 ± 0.61	4.61 ± 0.78*	4.72 ± 0.66^#^	0.003
LVDs (cm)	3.16 ± 0.78	3.32 ± 0.74	3.12 ± 1.03	0.805
IVS (cm)	1.23 ± 0.26	1.27 ± 0.25^†^	1.03 ± 0.23^#^	<0.001
PW (cm)	1.19 ± 0.24	1.23 ± 0.20^†^	1.00 ± 0.17^#^	<0.001
LVM (g)	245.1 ± 84.3	222.7 ± 71.2*^†^	173.4 ± 62.6^#^	<0.001
LVM index (g/m^2^)	155.9 ± 46.3	149.1 ± 50.8^†^	109.4 ± 31.8^#^	<0.001
Absolute LVM regression (g)	–	−30.7 ± 44.8	−76.8 ± 37.9	<0.001
LVM regression rate (%)	–	−7.2 ± 21.8	−30.0 ± 9.26	<0.001
Sinotubular junction (cm)	2.60 ± 0.37	2.62 ± 0.42	2.54 ± 0.40	0.558
Aortic cross sectional area (cm)	5.43 ± 1.59	5.52 ± 1.84	5.19 ± 1.70	0.558
LVEF (%)	58.8 ± 13.0	55.4 ± 11.1	56.5 ± 10.8	0.814

*LVDd* left ventricular diastolic diameter, *LVDs* left ventricular systolic diameter, *IVS* interventricular septal thickness, *PW* posterior wall thickness, *LVM* left ventricular mass, *LVEF* left ventricular ejection fraction

* Post-AVR (1 month) versus pre-AVR *P* value <0.05

^#^Post-AVR (12 months) versus pre-AVR *P* value <0.05

^†^Post-AVR (1 month) versus post-AVR (12 months) *P* value <0.05

**Table 3:** Change in severity of aortic stenosis (AS)

	Pre-AVR (*n* = 30)	Post-AVR (12 months) (*n* = 19)	*P* value
Peak aortic velocity (m/s)	4.55 ± 0.73	2.83 ± 0.59	<0.001
Mean pressure gradient (mmHg)	47.1 ± 16.0	14.5 ± 5.28	<0.001
EOA (cm^2^)	0.71 ± 0.18	1.42 ± 0.34	<0.001
EOAI (cm^2^/m^2^)	0.46 ± 0.18	0.91 ± 0.19	<0.001
ELCo (cm^2^)	0.83 ± 0.24	1.97 ± 0.58	<0.001
ELI (cm^2^/m^2^)	0.53 ± 0.15	1.26 ± 0.34	<0.001
ΔEOAI (cm^2^/m^2^)	–	0.41 ± 0.21	–
ΔELI (cm^2^/m^2^)	–	0.74 ± 0.37	–

*EOA* effective orifice area, *EOAI* effective orifice area index, *ELCo* energy loss coefficient, *ELI* energy loss index

**Fig. 1:**
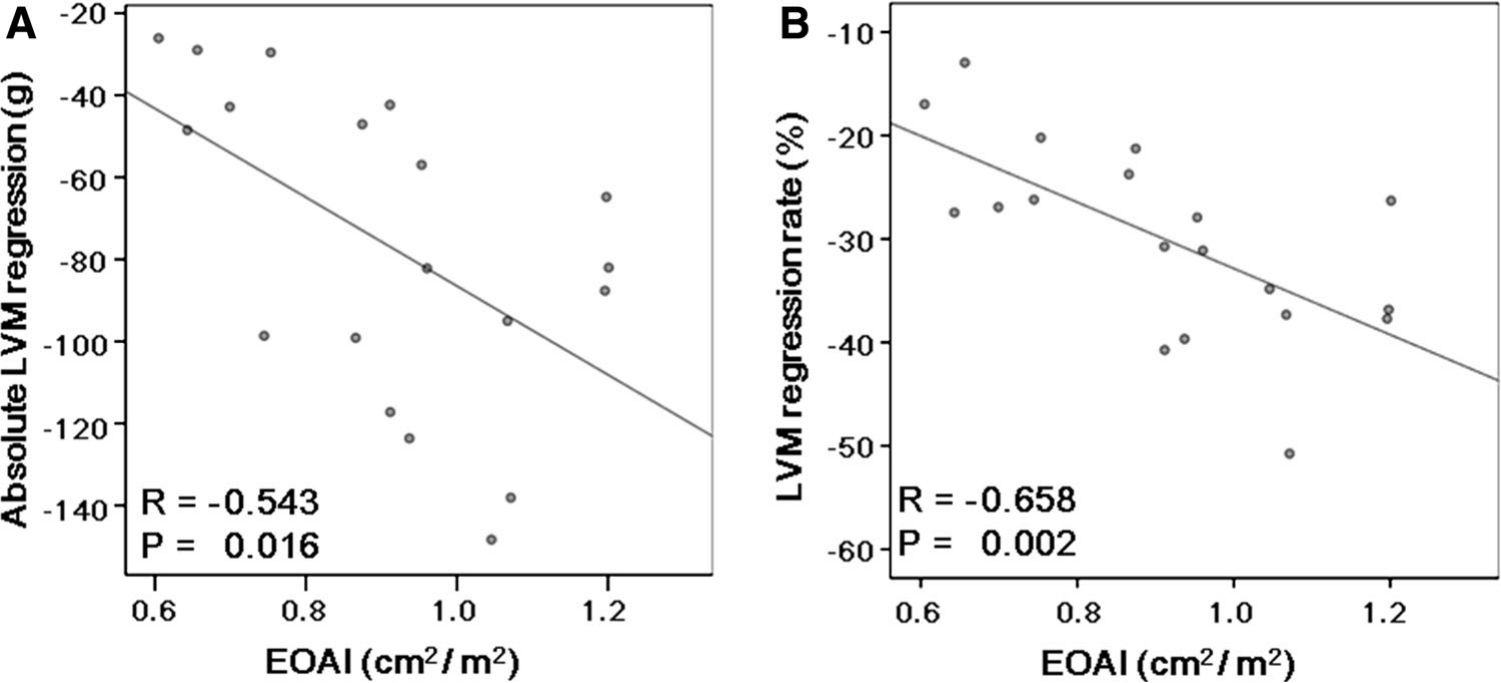
Comparison between the effective orifice area index (EOAI) after aortic valve replacement (AVR) and absolute left ventricular mass (LVM) regression (**a**) and the LVM regression rate (**b**). Both absolute LVM regression and the LVM regression rate correlated negatively with the EOAI

**Fig. 2:**
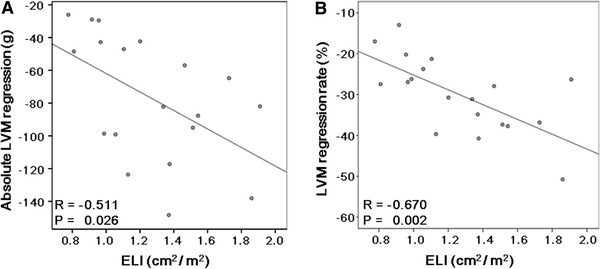
Comparison between the energy loss index (ELI) after AVR and absolute LVM regression (**a**) and the LVM regression rate (**b**). Both absolute LVM regression and the LVM regression rate correlated negatively with the ELI

**Fig. 3:**
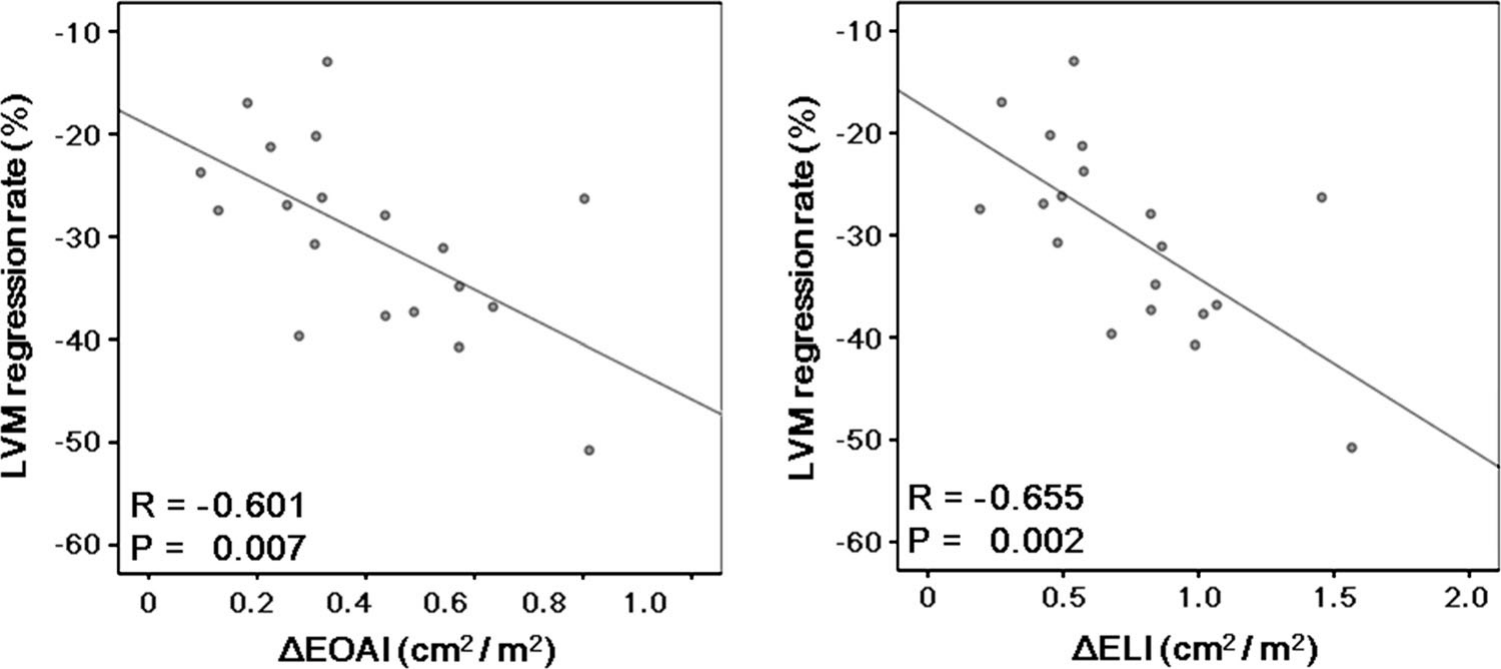
Comparison between the LVM regression rate and the increases in the effective orifice area index (ΔEOAI) or energy loss index (ΔELI) after AVR. Negative correlations were observed between the LVM regression rate and ΔEOAI (*R* = −0.601, *P* = 0.007) or ΔELI (*R* = −0.655, *P* = 0.002) after AVR

**Fig. 4:**
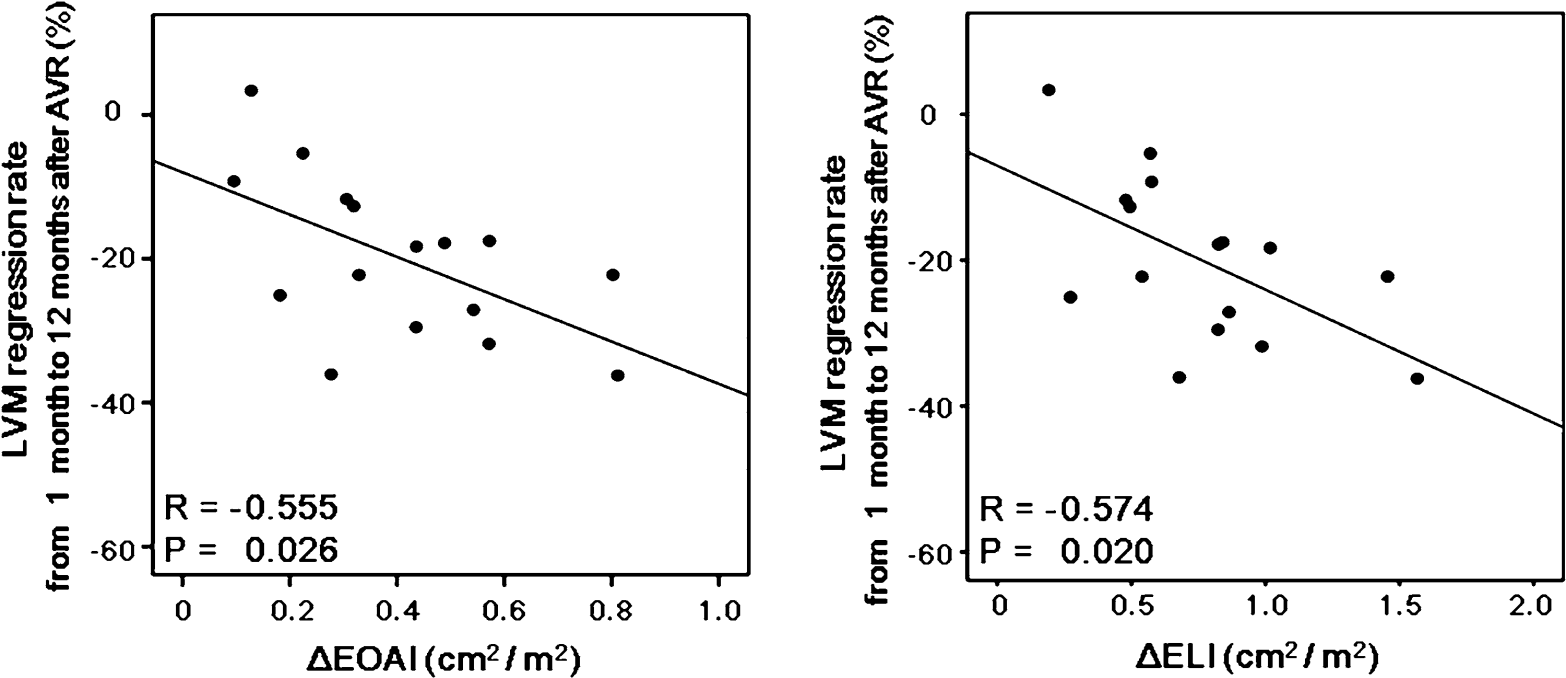
Comparison between the LVM regression rate from 1 to 12 months after AVR and the increases in the effective orifice area index (ΔEOAI) or energy loss index (ΔELI) after AVR. Negative correlations were observed between the LVM regression rate and ΔEOAI (*R* = −0.555, *P* = 0.026) or ΔELI (*R* = −0.574, *P* = 0.020) after AVR

There were no significant correlations between the peak aortic velocity after AVR and absolute LVM regression (*R* = −0.411, *P* = 0.080) or the LVM regression rate (*R* = −0.222, *P* = 0.360). On the other hand, negative correlations were observed between the post-operative EOAI and absolute LVM regression (*R* = −0.543, *P* = 0.016) or the LVM regression rate (*R* = −0.658, *P* = 0.002) (Fig. 1). Similarly, the post-operative ELI correlated negatively with absolute LVM regression (*R* = −0.511, *P* = 0.026) or the LVM regression rate (*R* = −0.670, *P* = 0.002) (Fig. 2). The LVM regression rate correlated negatively with both the ΔEOAI (*R* = −0.601, *P* = 0.007) and ΔELI (*R* = −0.655, *P* = 0.002) (Fig. 3). Similarly, the LVM regression rate from 1 to 12 months after AVR correlated negatively with both the ΔEOAI (*R* = −0.555, *P* = 0.026) and ΔELI (*R* = –0.574, *P* = 0.020) (Fig. 4). The mean value of the LVM regression rate was 30.0 %. Clinical characteristics and echocardiographic indices were compared between patients with smaller (<−30.0 %) and larger (≥−30.0 %) LVM regression rate (Tables 4, 5). There were no significant differences in the clinical characteristics between patients with smaller and larger LVM regression rates. Similarly, the pre-AVR echocardiographic indices did not differ between the 2 groups. On the other hand, the larger LVM regression group had significantly lower peak aortic velocity and mean pressure gradient and significantly larger ELI after AVR. By the ROC curve analysis, post-operative EOAI <0.91 cm^2^/m^2^ or post-operative ELI <1.12 cm^2^/m^2^ predicted smaller LVM regression rate (EOAI: area under curve = 0.799; *P* = 0.011 and ELI: area under curve = 0.825; *P* = 0.030, respectively).

**Table 4:** Clinical characteristics were compared between patients with smaller and larger left ventricular mass (LVM) regression rates

	Smaller LVM regression group (*n* = 10)	Larger LVM regression group (*n* = 9)	*P* value
Age (years)	63.8 ± 8.0	61.3 ± 5.4	0.437
Gender, male [*n* (%)]	6 (60)	4 (44)	0.656
Body surface area (m^2^)	1.58 ± 0.14	1.55 ± 0.25	0.765
Atrial fibrillation (%)	3 (30)	0 (0)	0.211
Angina [*n* (%)]	3 (30)	1 (11)	0.333
Syncope [*n* (%)]	0 (0)	0 (0)	–
Heart failure [*n* (%)]	7 (70)	5 (56)	0.650
Hypertension [*n* (%)]	6 (60)	7 (78)	0.628
Dyslipidemia [*n* (%)]	2 (20)	5 (56)	0.170
Diabetes mellitus [*n* (%)]	2 (20)	3 (33)	0.628
Smoking [*n* (%)]	4 (40)	5 (56)	1.000
Hemodialysis [*n* (%)]	4 (40)	5 (56)	0.656
Coronary artery disease [*n* (%)]	2 (20)	3 (33)	0.628

**Table 5:** Echocardiographic indices were compared between patients with smaller and larger LVM regression rates

	Pre-AVR		*P* value	Post-AVR (12 months)		*P* value
	Smaller LVM regression group (*n* = 10)	Larger LVM regression group (*n* = 9)		Smaller LVM regression group (*n* = 10)	Larger LVM regression group (*n* = 9)	
LVDd (cm)	5.16 ± 0.46	5.23 ± 0.79	0.828	4.931 ± 0.59	4.48 ± 0.69*	0.151
LVDs (cm)	3.35 ± 1.01	3.17 ± 0.70	0.667	3.37 ± 1.21	2.85 ± 0.76	0.282
IVS (cm)	1.17 ± 0.27	1.23 ± 0.16	0.588	1.01 ± 0.26*	1.05 ± 0.21*	0.746
PW (cm)	1.13 ± 0.31	1.21 ± 0.15	0.468	0.99 ± 0.21*	1.05 ± 0.10*	0.754
LVM (g)	243.9 ± 96.6	262.8 ± 82.1	0.578	183.1 ± 71.5*	162.6 ± 71.5*	0.492
LVM index (g/m^2^)	150.07 ± 52.9	166.7 ± 33.2	0.428	115.0 ± 39.7*	103.1 ± 20.4*	0.431
Absolute LVM regression (g)	–	–	–	−56.0 ± 27.9	−99.9 ± 34.8	0.007
LVM relative regression (%)	–	–	–	−23.0 ± 5.04	−37.7 ± 5.96	<0.001
Sinotubular junction (cm)	2.53 ± 0.30	2.75 ± 0.46	0.214	2.42 ± 0.37	2.64 ± 0.42	0.333
Aortic cross sectional area (cm)	5.07 ± 1.22	6.09 ± 2.05	0.216	4.68 ± 1.47	5.58 ± 1.86	0.347
LVEF (%)	53.0 ± 16.9	62.1 ± 9.1	0.316	54.8 ± 16.9	61.3 ± 8.34	0.472
Peak aortic velocity (m/s)	4.45 ± 0.55	4.60 ± 0.83	0.637	3.09 ± 0.45*	2.53 ± 0.60*	0.033
Mean pressure gradient (mmHg)	45.8 ± 16.8	47.8 ± 15.7	0.829	21.8 ± 7.35*	13.1 ± 6.02*	0.021
EOA (cm^2^)	0.69 ± 0.09	0.70 ± 0.16	0.921	1.29 ± 0.34*	1.60 ± 0.30*	0.049
EOAI (cm^2^/m^2^)	0.44 ± 0.06	0.46 ± 0.11	0.680	0.80 ± 0.18*	1.03 ± 0.11*	0.004
PPM [*n* (%)]	–	–	–	6 (60)	0 (0)	0.011
ELCo (cm^2^)	0.81 ± 0.12	0.80 ± 0.18	0.861	1.73 ± 0.58*	2.24 ± 0.46*	0.049
ELI (cm^2^/m^2^)	0.51 ± 0.07	0.52 ± 0.14	0.841	1.09 ± 0.34*	1.45 ± 0.24*	0.019
ΔEOAI (cm^2^/m^2^)	–	–	–	0.31 ± 0.20	0.52 ± 0.16	0.026
ΔELI (cm^2^/m^2^)	–	–	–	0.58 ± 0.35	0.93 ± 0.30	0.036

*LVDd* left ventricular diastolic diameter, *LVDs* left ventricular systolic diameter, *IVS* interventricular septal thickness, *PW* posterior wall thickness, *LVM* left ventricular mass, *LVEF* left ventricular ejection fraction, *EOA* effective orifice area, *EOAI* effective orifice area index, *PPM* prosthesis–patient mismatch (defined as EOAI <0.85 cm^2^/m^2^), *ELCo* energy loss coefficient, *ELI* energy loss index

* Post-AVR versus pre-AVR *P* value <0.05

**Fig. 5:**
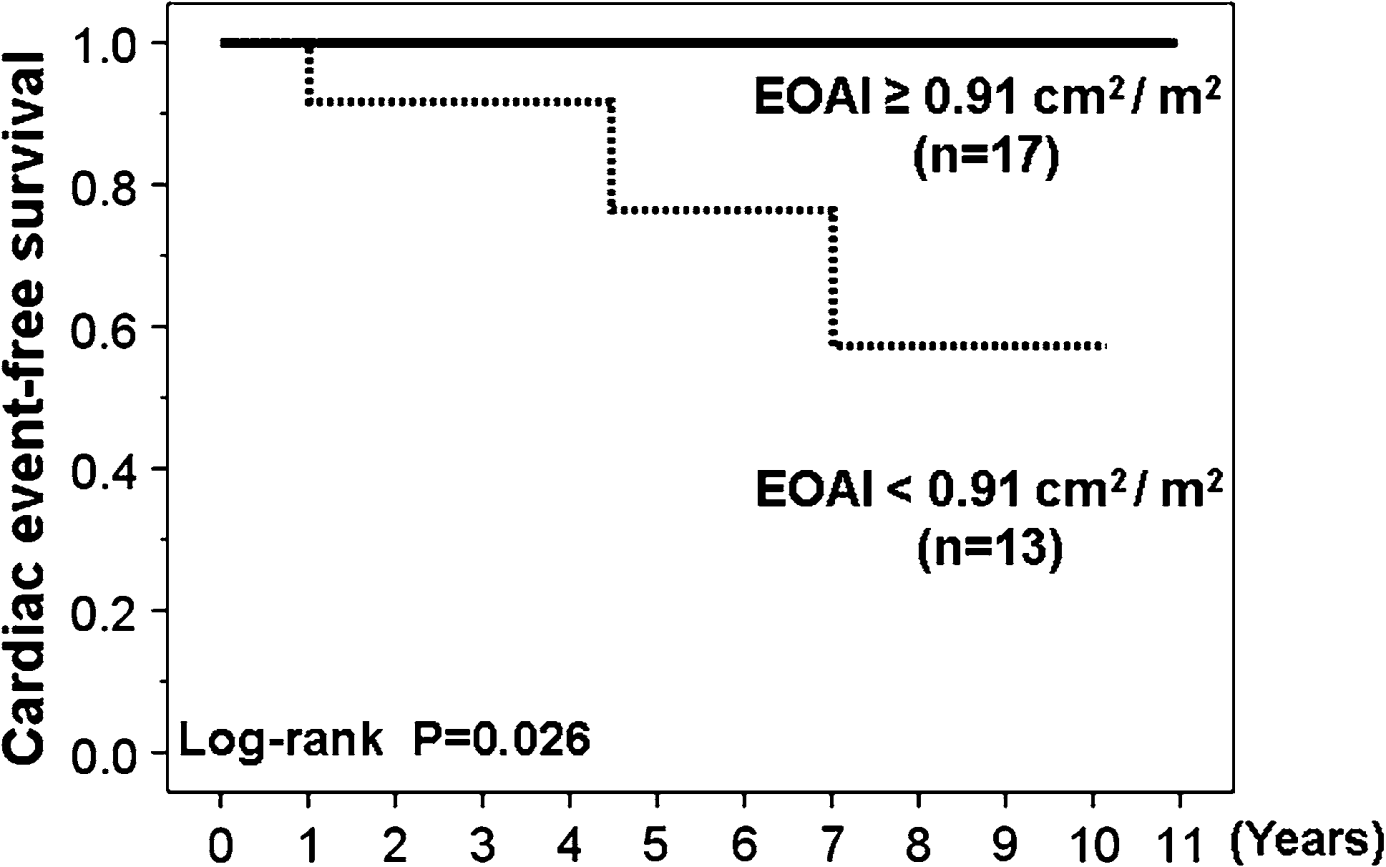
Kaplan–Meier curves comparing patients with effective orifice area index (EOAI) ≥0.91 cm^2^/m^2^ versus EOAI <0.91 cm^2^/m^2^. Event-free survival was significantly lower in patients with EOAI <0.91 cm^2^/m^2^

**Fig. 6:**
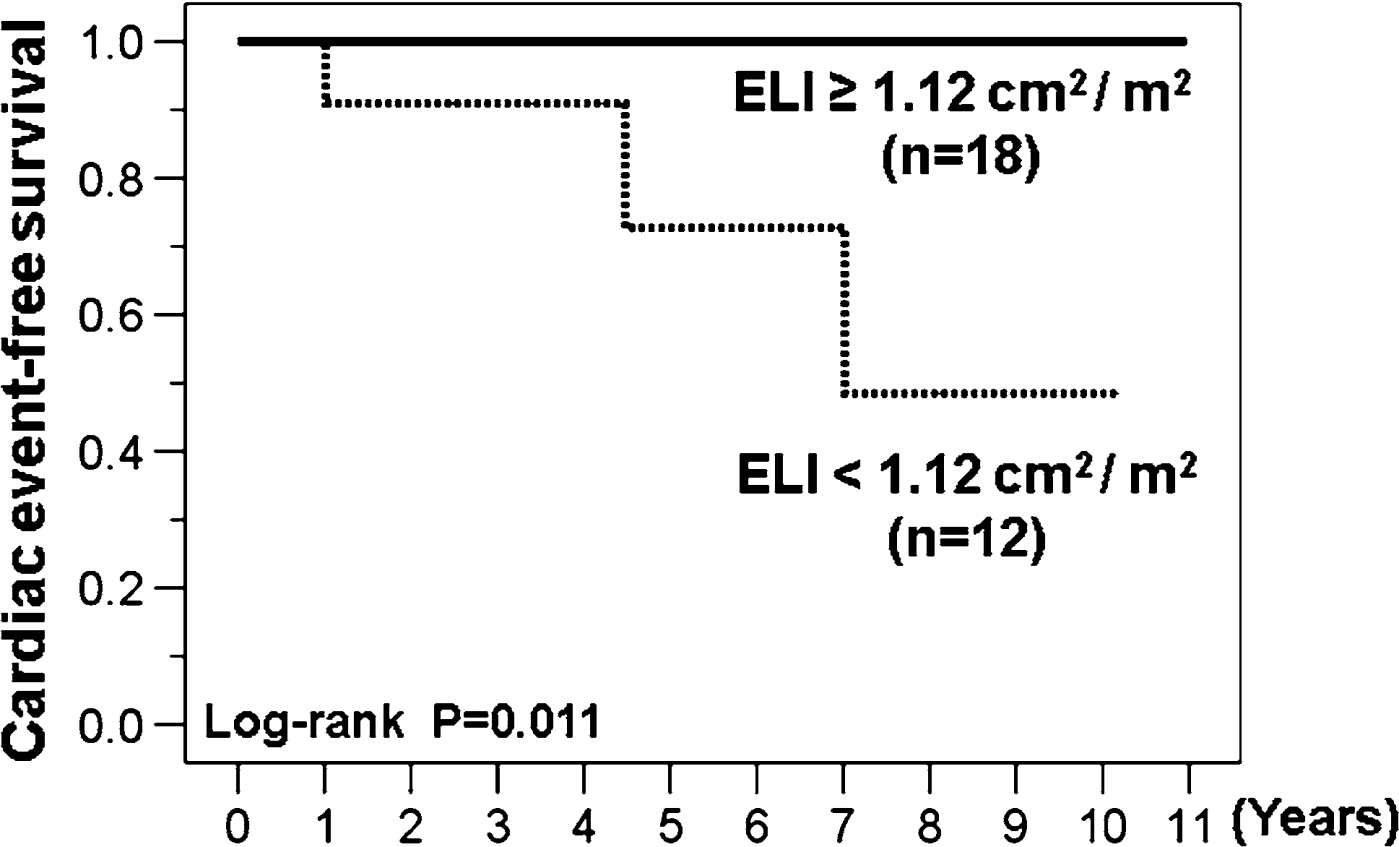
Kaplan–Meier curves comparing patients with energy loss index (ELI) ≥1.12 cm^2^/m^2^ versus ELI <1.12 cm^2^/m^2^. Event-free survival was significantly lower in patients with ELI <1.12 cm^2^/m^2^

During the follow-up period (median 5.2 years), patients with post-operative EOAI <0.91 cm^2^/m^2^ or post-operative ELI <1.12 cm^2^/m^2^ had significantly higher incidence of cardiac events (2 cardiac deaths and 1 heart failure) than patients with post-operative EOAI ≥0.91 cm^2^/m^2^ or post-operative ELI <1.12 cm^2^/m^2^. By Kaplan–Meier analysis, cardiac event-free survival was significantly lower in patients with post-operative EOAI <0.91 cm^2^/m^2^ or post-operative ELI <1.12 cm^2^/m^2^ than in patients with post-operative EOAI ≥0.91 cm^2^/m^2^ or post-operative ELI <1.12 cm^2^/m^2^ (Figs. 5, 6).

## Discussion

The main findings of this study were that: (1) the LVM regression rate was negatively and significantly correlated with the ELI, (2) ELI <1.12 cm^2^/m^2^ predicted smaller LVM regression rate (<−30.0 %) after AVR, and (3) patients with ELI <1.12 cm^2^/m^2^ had a higher incidence of cardiac events after AVR.

In our daily clinical settings, the peak transaortic flow velocity, mean pressure gradient, as well as the EOA derived from the continuity equation method are used to assess the severity of AS [23]. However, these measurements could be overestimated because of the pressure recovery phenomenon [11–13]. The concept of the pressure recovery phenomenon is based on fluid mechanics theory, showing that static pressure downstream of the stenosis could be increased or recovered because of the reconversion of kinetic energy into potential energy. Therefore, the peak or mean pressure gradient calculated from the maximal Doppler flow velocity could overestimate the true pressure gradient through the stenotic orifice. Recently, the ELCo or ELI has been proposed as a new Doppler-derived index to represent the functional severity of AS similar to the catheter-derived aortic valve area [11, 13, 18]. Previous studies have shown that the EOA in patients with AS can be corrected as the ELCo using the size of the ascending aorta [12, 13]. Several studies have documented that the Doppler-derived ELCo (or ELI) correlated better with the catheter-derived aortic valve area than the EOA (or EOAI) [11–13]. Interestingly, previous studies demonstrated that substantial numbers of patients who were initially diagnosed as severe AS based on the EOA may be re-categorized as moderate AS based on the ELCo [11, 24].

Pressure recovery may affect the assessment of the transprosthetic valvular pressure gradient, resulting in overestimation of the severity of prosthetic valvular stenosis [25, 26]. Aljassim et al. [27] reported that, even in patients with aortic prosthetic valves, the overestimation of the Doppler-derived indices can be predicted and corrected using the validated equation to calculate the ELCo in AS. Furthermore, our preliminary observation has shown that the ELCo predicts LVM regression in patients after AVR using bioprosthetic valves [14]. Because mechanical prosthetic valves have more complex orifice geometry as compared with bioprosthetic valves, it has not been well investigated whether the ELCo/ELI predicts LVM regression as well as prognosis. To the best of our knowledge, this is the first report to elucidate the significant relationship between the ELI and LVM regression after AVR with mechanical valves. In combination with previous reports and our present results, the ELI could be used as a functional index to assess LV pressure overload even after AVR and possibly be used as an index for predicting LVM regression after AVR with prosthetic valves [5, 11]. Although the LVM could be related to the severity of AS before AVR, indices of AS severity did not predict LVM regression after AVR, probably because AVR itself dramatically changes the severity of AS and, thus, pressure overload to the LV.

PPM is present when the inserted prosthetic valve is too small relative to the patient’s body size. PPM, defined as an EOAI ≤0.8 to 0.9 cm^2^/m^2^, has been shown to predict adverse outcomes [3–5, 7, 8, 14, 19, 22]. A recent meta-analysis of 34 observational studies including 27,186 patients showed a significant reduction in the overall and cardiac-related long-term survival for patients with PPM after AVR [28]. Theoretically, ELI the reflects LV pressure overload better than the EOAI.

In this study, 9 patients were diagnosed as classical PPM (defined as EOAI <0.85 cm^2^/m^2^). In 7 of 9 patients with EOAI <0.85 cm^2^/m^2^, the ELI was ≥0.85 cm^2^/m^2^. The LV mass regression after AVR was numerically greater in patients with ELI ≥0.85 cm^2^/m^2^ than in patients with ELI <0.85 cm^2^/m^2^ (−30.9 ± 9.2 vs. −22.2 ± 7.4 %), although the difference could not be statistically tested because of the small sample size. However, impact of the ELI on clinical events after AVR with mechanical valves has not yet been clarified. Although ELI <1.12 cm^2^/m^2^ had more cardiac events after AVR in our present study, it is still inconclusive as to whether the ELI is a stronger predictor of cardiac events than the EOAI because of the small sample size and relatively lower events rates in our current study population.

## Limitations

The main limitation of this study is that this is a retrospective, single-center study with a small sample size. As mentioned in the discussion, the impact of the ELI on the clinical outcome might be affected by possible selection bias. In fact, 37 % of our current study population comprised chronic renal failure patients on hemodialysis, who were known to have a very high risk for operative and late mortality [29]. Therefore, this study may be underpowered to be generalized to all AS patients.

Another limitation of this study is the possible change in aortic diameter after AVR. Botzenhardt et al. [30] reported that aortic diameters decreased after removal of the diseased valve. Therefore, changes in aortic diameter after AVR might have affected the results. Different kinds of mechanical prosthetic valves have their own flow property, although all valves analyzed in this study were bi-leaflet mechanical valves. Therefore, these differences in the prosthetic valve type might have affected the results of our study.

## Conclusions

The energy loss index (ELI) as well as the effective orifice area index (EOAI) could predict left ventricular mass (LVM) regression after aortic valve replacement (AVR) with mechanical valves. Whether the ELI is a stronger predictor of clinical events than the EOAI is still unclear, and further large-scale study is necessary to elucidate the clinical impact of the ELI in patients with AVR.
